# PFAPA syndrome as an hereditary autoinflamatory disorder

**DOI:** 10.1186/1546-0096-13-S1-P198

**Published:** 2015-09-28

**Authors:** C Kadhim, F Maiolini, L Cerrito, LL Sicignano, M Giovinale, E Verrecchia, F Gurrieri, M Genuardi, R Manna

**Affiliations:** 1 Catholic University of the Sacred Heart, Internal Medicine, Rome, Italy; 2 Catholic University of the Sacred Heart, Human Genetics, Rome, Italy

## Introduction

PFAPA syndrome (periodic fever, aphtous stomatitis, pharyngitis, adenitis) is an autoinflammatory disease, for which no genetic marker has been identified yet, and its etiology remains unknown. However, the clinical and biochemical similarities to other autoinflammatory conditions, including Familial Mediterranean Fever (FMF), suggest that a genetic impairment might constitute the underlying cause of the disease. FMF is the most widespread monogenic autoinflamatory disorder. In 60% of patients affected by FMF two concurrent mutations of MEFV gene have been demonstrated, whereas in 30% one mutation of the same gene has been shown. In only 10% of patients, no genetic marker has been identified.

## Objectives

Our study stems from the hypothesis that PFAPA and FMF MEFV-negative (MEFVneg) patients might share a genetic marker accounting for the development of signs and symptoms of the disease. In these patients, a careful familiar history and the presence of accompanying symptoms throughout the flares were investigated.

## Materials and methods

We have been performing a cohort study, involving 67 MEFVneg patients and 51 PFAPA patients. These populations have been compared in terms of clinical manifestations and evidence of periodic fever and surgical tonsillectomy in parents.

## Results

A substantial overlap of clinical manifestations has been observed in the two cohorts. Patients affected by PFAPA frequently presented with abdominal (49%), articular (64%), thoracic pain (14%). On the other hand, MEFVneg patients showed aphthosis (58%), pharyngitis (55%) and adenopathies (49%). Moreover in 58% of PFAPA patients a history of periodic fever in one or both parents during childhood was demonstrated. Tonsillectomy was performed in 51% of the parents of PFAPA patients. In MEFVneg patients, on the other hand, the parents with a history of periodic fever during childhood were 32%, whereas the amount of tonsillectomies reached up to 28%.

## Conclusion

These findings unveil the possibility that PFAPA might be a genetic disease, whose pathogenesis recapitulates the hereditary transmission pattern already observed in MEFV-positive FMF or other autoinflammatory disorders. This hypothesis clearly sheds the light on the need to identify the gene(s) involved in the activation of the inflammasome and, hence, in the development of the disease. Furthermore, due to high clinical affinity to FMF, such a genetic signature for PFAPA might potentially result as useful to account for MEFV-negative FMF.

